# Novel Compound Heterozygote Variations in FADD Identified to Cause FAS-Associated Protein with Death Domain Deficiency

**DOI:** 10.1007/s10875-020-00779-6

**Published:** 2020-04-29

**Authors:** Lisa A. Kohn, Joseph D. Long, Edward C. Trope, Caroline Y. Kuo

**Affiliations:** Department of Pediatrics, Division of Allergy, Immunology and Rheumatology, University of California, Los Angeles, Los Angeles, CA USA

**Keywords:** FADD deficiency, Autoimmune lymphoproliferative syndrome, Genetics, Apoptosis

To the Editor

Autoimmune lymphoproliferative syndromes (ALPS and related disorders) are characterized by insufficient apoptosis due to defects in the FAS apoptosis pathway. FADD deficiency (OMIM: 602457; first described in Bolze et al. 2010 [1]) is an autosomal recessive disorder resulting from a pathogenic variation in *FADD* (FAS-associated protein with death domain), the adaptor protein involved in Fas signaling to caspases 8 and 10. Previously described FADD deficiency patients demonstrate a clinical phenotype partially overlapping with other ALPS disorders (decreased Fas-mediated lymphocyte apoptosis, increased circulating double negative T cells, elevated soluble Fas ligand, IL-10, and vitamin B12) but also recurrent febrile episodes with encephalopathy and seizures, variable degrees of lymphadenopathy or splenomegaly, cerebral atrophy, and structural cardiac abnormalities. Recurrent severe viral infections are thought to be mediated by FADD’s role in TLR-independent innate immune responses and induction of IRF7 and IFN-α [2]. There are very few cases in the literature of FADD deficiency patients (4 patients from a consanguineous family in the original report [1] and 2 patients in a second report [3]). These patients seem to have worse outcomes than classical ALPS patients. Three of four patients from the original report died prior to 5 years old from invasive pneumococcal infections or death during episodes of encephalopathy. In the second report, multiple family members who were presumed to be affected had also died in early childhood, and both patients described in that report underwent hematopoietic stem cell transplantation. All prior reports of patients with FADD deficiency were homozygous for the pathogenic variation p.C105W. Here, we present a case of FADD deficiency identified by clinical exome sequencing with novel compound heterozygote genetic variations whose clinical presentation is consistent with prior described cases.

At 14 months of age, our unvaccinated patient presented with fever, rash, vomiting, and status epilepticus with respiratory arrest that required intubation. He had enlarged cervical lymph nodes that regressed with antibiotics and steroids. He recovered from this initial episode but subsequently experienced a series of similar illnesses with fevers, altered mental status, and seizures. With the exception of elevated serum HHV6 IgG, extensive infectious workup up was negative (Supplemental Table 1). His family history was significant for an older brother with an episode of fever and status epilepticus requiring intubation 5 days after his first MMR vaccination. He experienced similar episodes until his demise at 18 months old (Fig. 1).

**Fig. 1:**
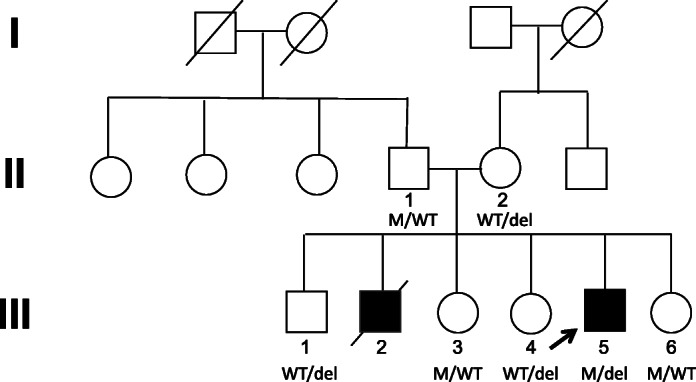
Pedigree of family. Affected individuals shaded black, deceased individuals with diagonal line. Arrow indicates proband. WT: wild type; M: missense mutation; del: deletion mutation

Our patient’s clinical and laboratory findings were similar to previously described FADD deficiency patients. Physical exam was notable only for a reticulated, lacy appearance to his skin. His CBC was normal except for an elevated absolute lymphocyte count (Supplemental Table 2). The absolute CD3+ count was elevated with an increased percentage of double-negative CD3+ TCRαβ+ CD4− CD8− cells (Supplemental Table 3). He had elevated absolute CD19+ B cells but normal levels of IgG, IgA, and IgM for age (Supplemental Tables 4, 5). Vaccine titers could not be assessed as he was completely unvaccinated, but he lacked appropriate isohemagglutinins. T cell proliferative responses to mitogen and antigen stimulation were normal, with the exception of low B cell proliferation in response to pokeweed (on two occasions) and lack of vaccine antigen (tetanus)-induced proliferation, which is consistent with his unvaccinated status (Supplemental Table 5). Soluble Fas ligand, IL-10, IL-18, and vitamin B12 levels were increased, while his IFN-γ and IL-1β levels were normal (Supplemental Tables 5, 6). There was no hepatosplenomegaly detected by MRI or ultrasound, but Howell Jolly bodies were increased in the peripheral blood indicating functional hyposplenism, and he has intermittently had mild elevations of ALT and AST. He has not had cytopenias or autoimmunity. Testing for Fas mediated apoptosis showed a profound deficiency in appropriate cell death (Fig. 2 and Supplemental Table 7). No abnormalities were seen on echocardiogram. The patient had mild neurodevelopmental delay, and MRI of the brain showed restricted diffusion of the white matter of the corpus callosum. He was recently diagnosed with retinal ischemia with vitriol hemorrhages and will undergo laser repair.

**Fig. 2:**
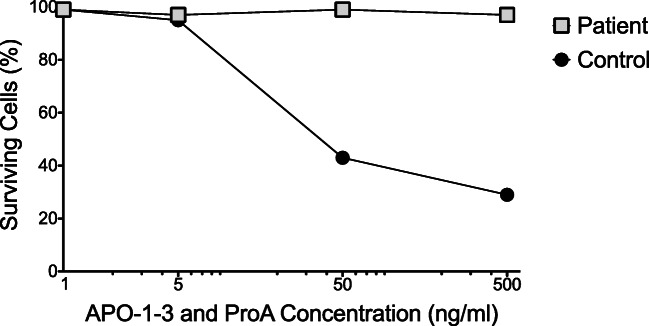
Fas-mediated apoptosis. Patient and control peripheral blood mononuclear cells are activated with Concanavalin A prior to expansion in culture. Expanded T cells are then treated with agonist anti-Fas antibody (APO-1-3) and protein A in the presence of IL-2 to evaluate Fas-mediated lymphocyte apoptosis. After treatment, cells are stained with propidium iodide and analyzed by flow cytometry to determine viability. Performed at Cincinnati Clinical laboratories

Whole-exome sequencing revealed two different genetic alterations in the *FADD* gene: a maternally inherited nonsense variant characterized by a 7 base-pair deletion (c.52_58delGACGAGC) predicted to severely truncate the protein, and a paternally inherited rare missense variant c.313 T > C (p.C105R) (Figs. 1 and 3). The maternally inherited deletion is not reported in gnomAD, and the paternally inherited missense variation has a mean allele frequency < 0.001% in gnomAD with no reported homozygous individuals, making both variants extremely rare. Although this specific missense variation has not previously been described as pathogenic, this nucleotide encodes a highly conserved residue in the FADD death domain and a different variant in the same nucleotide of *FADD* (c.315 T > G) has been associated with FADD deficiency [1]. Pathogenicity predictions characterized the missense mutation as probably damaging (PolyPhen) or deleterious (SIFT). FADD protein levels as measured by immunoblot (Cell Signaling Technology 2782S, Danvers, MA) revealed lower FADD protein levels in primary T cells from the compound heterozygote proband (Lane 6) compared to a healthy control (Lane 8) (Figs. 4 and 5). Protein levels from healthy family members heterozygous for either variant were lower than the healthy control, indicating an alteration in gene dosage without an obvious clinical phenotype. Apoptosis and soluble Fas ligand levels were measured in relatives heterozygous for either the missense mutation or the deletion. Heterozygous members were shown to be either normal or mildly abnormal, but not as severely affected as the compound heterozygote proband (Supplemental Tables 7 and 8).

**Fig. 3:**
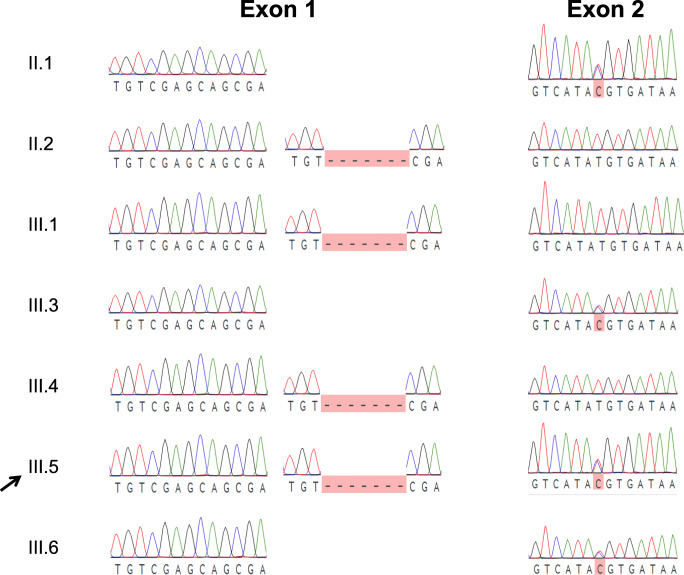
Sequencing of proband (arrow) and immediate family. PCR fragments were amplified from genomic DNA, cloned into a plasmid backbone (TOPO TA cloning; ThermoFisher cat. no 450071), followed by Sanger sequencing of multiple clones. Proband is a compound heterozygote with a nonsense variant due to a 7 bp deletion inherited from his mother and a missense variant from his father. Four living siblings have confirmed heterozygosity for one of two variants

**Fig. 4:**
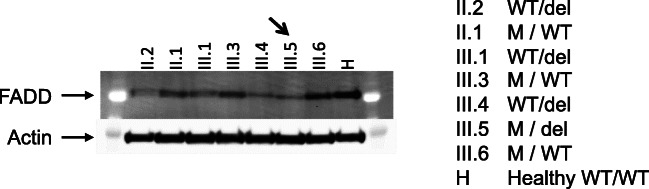
FADD immunoblot of primary T cells from patient, relatives, and control, with equal amounts of cell lysate loaded per well. Actin loading control. WT: wild type; M: missense mutation; del: deletion mutation. FADD polyclonal antibody against residue Ser194 (Cell Signaling Technology 2782); FADD secondary antibody (Anti-mouse IgG HRP-linked Antibody, Cell Signaling Technology 7076S); Pierce ECL Plus Western Blotting Substrate (ThermoFisher no. 32134); Actin secondary antibody (AF647 goat anti-mouse IgG Invitrogen A21236)

**Fig. 5:**
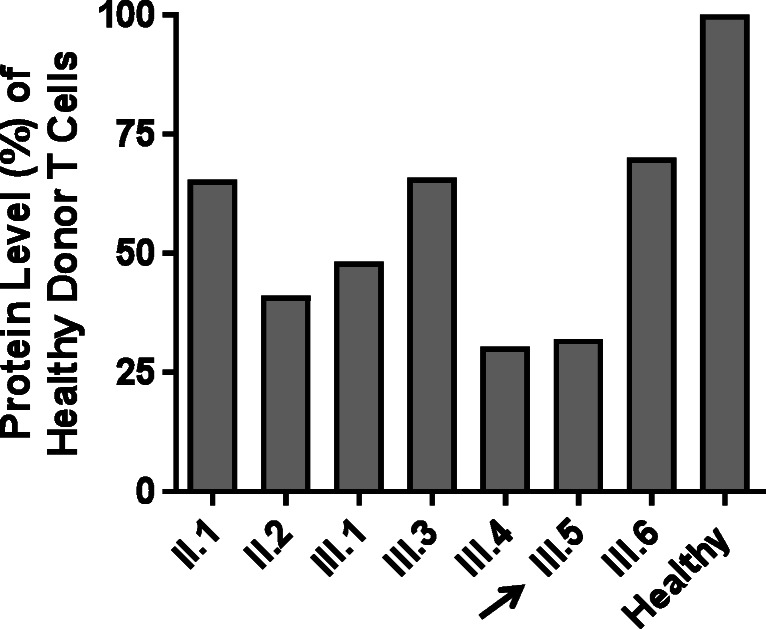
FADD immunoblot protein levels in primary T lymphocytes normalized to actin control as measured by densitometry

Due to a family history of intractable seizures in the older brother after MMR vaccination, our patient is completely unvaccinated and remained so despite recommendations for inactivated vaccines. However, given his functional hyposplenism and risk of sepsis from encapsulated organisms, we have recommended to the patient’s family antibiotic prophylaxis to prevent death from pneumococcal sepsis. The family declined antibiotic prophylaxis, although there are ongoing discussions continuing to recommend it. To address possible impaired viral responses and lack of appropriate B cell antibodies (isohemagglutinins), we initiated subcutaneous immunoglobulin infusions at 400 mg/kg/month. The patient tolerated these infusions well with no side effects and subjective improvements in fatigue and decreased respiratory infections overall for the past 8 months.

Of the FADD deficiency patients described in the literature, several died prior to 5 years old, as did our patient’s sibling who likely had the same compound heterozygous variations as the proband. However, the natural history and optimal interventions for FADD deficiency patients remain to be determined, as so few prior patients have been described. Two previously described patients underwent hematopoietic stem cell transplantation (HSCT) [3]. The first patient experienced multiple post-transplant morbidities including cytopenias, enteropathies, and vasculopathy, while the second patient tolerated transplant with minimal morbidity [3]. Our patient is undergoing evaluation with our primary immune deficiency transplantation team to determine if he is an appropriate transplant candidate. The transplant evaluation will consider the abnormal quantification of Fas-mediated apoptosis in each of the family members, all of whom are heterozygous for one of the mutations with decreased FADD protein expression despite appearing phenotypically normal, when determining if family members would be a good match. HSCT will likely be an important part of the treatment regimen for FADD deficiency, but given the rarity of the disease and the paucity of data on all of the potential clinical manifestations, it remains unclear what degree of donor chimerism is needed to be curative of the immunodeficiency and how HSCT will affect non-hematopoietic manifestations of FADD deficiency.

## Electronic Supplementary Material

ESM 1 (PDF 84 kb)
