# Supplementary material for: Novel Compound Heterozygote Variations in FADD Identified to Cause FAS-Associated Protein with Death Domain Deficiency
Source: J Clin Immunol. 2020 Apr 29;40(4):658–61. doi: 10.1007/s10875-020-00779-6 (PMC7253512; doi:10.1007/s10875-020-00779-6)
Supplement: Supplementary file 1 — (PDF 84 kb) [file 10875_2020_779_MOESM1_ESM.pdf]

Supplemental Table 1

**Test**	**Patient Value**	**Reference Range**
HHV6 IgG	*Positive*	Negative
CMV IgG	Negative	Negative
CMV PCR	Negative	Negative
EBV-EA IgG	Negative	Negative
EBV VCA IgG	Negative	Negative
EBV VCA IgM/EBNA IgG	Negative	Negative
EBV PCR	Negative	Negative
Parvovirus B19 IgM	Negative	Negative
Parvovirus B19 IgG	Negative	Negative

Supplemental Table 2

**Test**	**Patient Value**	**Reference Range**
Hemoglobin	12	11.5-15.5 (g/dL)
Hematocrit	37.2	35-45 %
Platelets	321	143-398 (x10^3 µL)
WBC	18.5	5-14.5 (x10^3 µL)
ANC	4,600	1.5-8 (x10^3 µL)
Total Lymphocytes	*8,740 (H)*	1.5-7(x10^3 µL)
CD3+ (T cells)	*5,947 (H)*	603-2990/cmm
CD16+CD56+ (NK)	207	95-640/cmm
CD19+ (B cells)	*2,186 (H)*	107-698/cmm

Supplemental Table 3

Test	Patient Value Total, (%)	Reference Range Total, (%)
Total B cells CD19+	2,014 (27%)	107-698/cmm (7-27%)
Memory B (CD19+CD27+)	137 (6.8%)	18-145/cmm (6-53%)
IgM Memory B cells (CD19+CD27+IgM+)	6 (0.3%)	0-12/cmm (0-5%)
Unswitched Memory B (CD19+CD27+IgM+IgD+)	62 (3.1%)	4-85/cmm (2-29%)
Switched Memory B (CD19+CD27+IgM-IgD-)	56 (2.8%)	7-61/cmm (2-27%)
Immature B cells (CD19+CD21Low)	107 (5.3%)	0.3-22/cmm (0.2-9%)

Supplemental Table 4

Test	Patient Value	Reference Range
T-Lymph (CD3+)	*5,271* (70%)	603-2,990 (49-84%)
T-Helper (CD4+)	*2,748* (36%)	441-2,156 (28-63%)
T Cytotoxic (CD8+)	*2,065* (27%)	125-1,312 (10-40%)
Ratio (CD4:CD8)	1.33	0.96-3.93
CD4 Naïve (CD4+CD45RA+)	*1,374* (50%)	84-563 (22-53%)
CD Memory (CD4+ CD45RO+)	*1,209* (44%)	204-696 (34-74%)
CD4 CD62L Naive (CD4+CD45RA+CD62L+)	*1,347* (49%)	72-717 (26-64%)
CD4 Effector Memory (CD4+CD45RA-CD62L-)	*550* (20%)	34-244 (9-24%)
CD4 Central Memory (CD4+CD45RA-CD62L+)	*769* (28%)	138-484 (22-53%)
CD4 TEMRA (CD4+CD45RA+CD62L-)	*82* (3%)	0-39 (0-4%)
CD8 Naïve (CD8+CD45RA+)	*1,693* (82%)	74-530 (42-73%)
CD8 Memory (CD8+CD45RO+)	*330* (16%)	53-258 (14-47%)
CD8 CD62L Naïve (CD8+CD45RA+CD62L+)	*1,466* (71%)	23-474 (22-71%)
CD8 Effector Memory (CD8+CD45RA-CD62L-)	*289* (14%)	26-172 (8-32%)
CD8 Central Memory (CD8+CD45RA-CD62L+)	*83* (4%)	6-11 (2-21%)
CD8 TEMRA (CD8+CD45RA+CD62L-)	*248* (12%)	0-233 (0-43%)
TCR αβ+	*5597* (64%)	1253-2216 (59-81%)
TCRαβ+ CD4-CD8- (Double Negative T cells)	*411 (4.7%)*	11-37 (0.3-1.7%)
HLADR+ activated	*833* (14%)	51-246 (3-25%)
CD3+CD25+/HLA DR	1.6	1.1-10.6

Supplemental Table 5

**Test**	**Patient Value (mg/dL)**	**Reference Range (mg/dL) Based on Caliper, Age specific**
IgG	1,068	504-1464
IgA	204	50-240
IgM	27	40-140
Isohemagglutinins (Blood type A+)		
A1	<1:2	
A2	<1:2	
B	*<1:2*	
**Vaccine titers**		
Tetanus	0.5	>0.1 protective
S. pneumoniae	0/23	17/23 protective
H. Influenzae B	4.0	>1.0 protective

**Test**	**Patient Value**	**Reference Range**
CH50	111	60-144 CAE Units
AH50	94% of normal	>46

**Lymphocyte Proliferation**<br>Patient Value [Reference Range]					
	Lymphs	CD3	CD4	CD8	CD19
PHA	85 [>33]	89 [>41]	82 [>28]	96 [>58]	
Concanavalin A	65 [>29]	71 [>35)	78 [>34]	62 [>34]	
Pokeweed	28 [>10]	35 [>2]	21 [>1]	47 [>5]	*2* [>11]
Tetanus	0 [>2]	0 [>1]	0 [>2]		
Candida	64 [>4]	60 [>2]			

**Test**	**Patient Value**	**Reference Range**
Cytotoxic T Lymphocyte assay	Normal	Normal

Supplemental Table 6

**Test**	**Patient Value**	**Reference Range**
Vitamin B12	*1415 pg/ml*	254-1060 pg/ml

**Test**	**Patient Value**
Peripheral smear findings	*+Howell Jolly bodies*
ANA	Negative

**Test**	**Patient Value (pg/ml)**	**Reference Range (pg/ml)**
IL1β	5	≤ 25
IL2	3	≤3
IL4	5	≤8
IL6	3	≤6
IL10	*9*	≤6
IL18	*591*	89-540
IFNg	1	≤5
TNFa	1	≤7

Supplemental Table 7

	II.1 M/WT	II.2 WT/del	III.1 WT/del	III.3 M/WT	III.4 WT/del	III.5 M/Del (Proband)	III.6 M/WT	Reference Range
Apoptosis	77%	72%	*44%* (L)	74%	*56%* (L)	*2%* (L)	*58%* (L)	68-93%
Serum FasL (pg/ml)	147	129	*274* (H)	184	*233* (H)			37-226
						758 (H)	271	70-308

Supplemental Table 7: Fas-mediated apoptosis and serum FasL levels.

Supplemental Table 8

ALPs Testing	II.1 (M/WT)		II.2 (WT/del)		III.5 (M/del) (Proband)		Reference	
	**Patient Value**	**Positive/ Negative**	**Patient Value**	**Positive/ Negative**	**Patient Value**	**Positive/ Negative**	**Reference Range**	**Criteria**
TCRαβ+ CD4-CD8- (Double Negative T cells)	NR (1.3%)	Negative	16 (0.8%)	Negative	*411 (4.7%)*	Positive	11-37 (0.3-1.7%)	>2% or >68 cells/ mcl
B220 TCR a/b DNTCs >60%	22%	Negative	16%	Negative	*61%*	Positive	5-25%	>60%
CD3+CD25+/HLA DR Ratio	3.4	Negative	4.7	Negative	1.6	Negative	1.1-10.6	<1.0
CD27+ B cells <15%	68%	Negative	24%	Negative	*6%*	Positive	11-51%	<15%
Total Score	0/4		0/4		3/4			

Lymphocyte Proliferation	II.1 (M/WT)	II.2 (WT/del)	Reference
	**Patient Value**	**Patient Value**	**Reference Range**
PHA	236,195	258,199	≥135,000
Tetanus	8,273	*1,753*	≥4,761
Candida	246,973	96,263	≥15,289

Supplemental Table 8: ALPs testing and lymphocyte proliferation assays. NR (Not reportable) - Absolute values for II.1 not available due to lack of simultaneous CBC with differential. Testing performed by Cincinnati Children’s Hospital Medical Center Cancer and Blood Diseases Institute – Diagnostic Immunology Laboratory
